# The Reelin Receptors Apolipoprotein E receptor 2 (ApoER2) and VLDL Receptor

**DOI:** 10.3390/ijms19103090

**Published:** 2018-10-09

**Authors:** Paula Dlugosz, Johannes Nimpf

**Affiliations:** Department of Medical Biochemistry, Max F. Perutz Laboratories, Medical University Vienna, 1030 Vienna, Austria; Paula.Dlugosz@meduniwien.ac.at

**Keywords:** apolipoprotein E receptor 2, VLDL receptor, reelin, clusterin, Alzheimer’s disease

## Abstract

Apolipoprotein E receptor 2 (ApoER2) and VLDL receptor belong to the low density lipoprotein receptor family and bind apolipoprotein E. These receptors interact with the clathrin machinery to mediate endocytosis of macromolecules but also interact with other adapter proteins to perform as signal transduction receptors. The best characterized signaling pathway in which ApoER2 and VLDL receptor (VLDLR) are involved is the Reelin pathway. This pathway plays a pivotal role in the development of laminated structures of the brain and in synaptic plasticity of the adult brain. Since Reelin and apolipoprotein E, are ligands of ApoER2 and VLDLR, these receptors are of interest with respect to Alzheimer’s disease. We will focus this review on the complex structure of ApoER2 and VLDLR and a recently characterized ligand, namely clusterin.

## 1. Introduction

**Figure 1:**
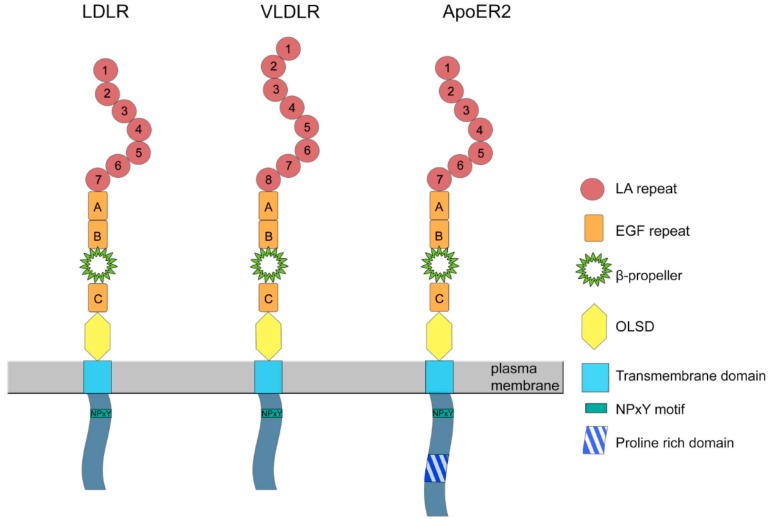
Structures of low density lipoprotein receptor (LDLR), VLDL receptor (VLDLR), and Apolipoprotein E receptor 2 (ApoER2). The LDL receptor and its closest relatives VLDL receptor and ApoER2 are composed of six characteristic superimposable structural modules: (1) ligand binding domain with a variable number of LDL receptor type A repeats (LA repeats), (2) epidermal growth factor precursor-like repeats A, B, and C (EGF repeats), (3) a YWTD containing domain forming a so-called β-propeller, (4) the *O*-linked sugar domain (OLSD), (5) a transmembrane domain, and (6) a cytoplasmic domain containing the NPXY motif and facultatively a proline-rich domain unique for ApoER2. Domains are not drawn to scale.

Apolipoprotein E receptor 2 (ApoER2) and VLDL receptor (VLDLR) belong to the low density lipoprotein receptor (LDLR) family, a class of type-I transmembrane receptors with high homology to their name-giving member the LDL receptor. Besides more distant members of this family, such as LRP 1, 1b, 2, 5, and 6; ApoER2, VLDLR, and LDLR have a superimposable structure indicating that the corresponding genes may have evolved from one single ancestor by gene duplication events and minor exon rearrangements. The architecture of these proteins (Figure 1) is characterized by six structural modules: (1) A N-terminal ligand binding domain with a variable number of LDL receptor type A repeats (LA repeats), (2) an invariant number of three epidermal growth factor (EGF) precursor-like repeats (type B repeats), (3) a YWTD β-propeller, (4) an *O*-linked sugar domain (OLSD), (5) a transmembrane domain, and (6) a cytoplasmic domain containing the NPXY motif that mediates clathrin-mediated endocytosis of these receptors but also the interaction with other proteins rendering ApoER2 and VLDLR to signal transduction receptors as well. Another common denominator of these three receptors is the fact that all of them bind apolipoprotein E. In sharp contrast to the LDLR, which has a very specific function in mediating cellular and systemic cholesterol homeostasis, ApoER2 and VLDLR have many unrelated ligands and have common and/or different functions depending on the species/organs/cells they are expressed in and depending on the ligand(s) present in the particular setting. Their role in the Reelin signaling pathway was nicely demonstrated in mice lacking ApoER2 and VLDLR. The loss of both receptors leads to a Reeler/Disabled-like phenotype in mice that is characterized by changes in the architecture of laminated structures in the brain [1,2]. 

## 2. Structure and Expression of ApoER2

ApoER2 was originally discovered in human [3] and chicken [4]. The human transcript comprises an open reading frame of 2889 bp encoding a protein with 963 amino acids and a molecular weight of 105 kDa. The sequence identity at the protein level between ApoER2 and LDLR is 49% and between ApoER2 and VLDLR around 47%, demonstrating the close relationship between these three proteins. ApoER2 is preferentially expressed in the central nervous system (neocortex, cerebellum, hippocampus, and olfactory bulb [5]) and to a minor extent also in the peripheral nervous system (sciatic nerve and Schwann cells) [6]. In cortical neurons, ApoER2 is expressed in apical dendrites and cell bodies; in Purkinje cells of the cerebellum in the most distal dendritic processes; in the hippocampus, ApoER2 is found in pyramidal cells of all CA subfields and granule cells of the dentate gyrus [7]. In addition, ApoER2 is expressed by radial glia and intermediate progenitor cells (IPCs) [8]. Outside the nervous system, ApoER2 can be found in placenta, testis, ovary [3], and platelets [9].

Since the best characterized function of ApoER2 is its involvement in the Reelin pathway, its expression in the murine central nervous system during embryonic development was studied in close detail [10,11]. Immunocytochemistry and in situ hybridization studies revealed that ApoER2 is predominantly expressed in the lower part of the intermediate zone (IZ) and in the multipolar accumulation zone (MAZ). Moderate levels of ApoER2 were found in the upper part of the marginal zone (MZ). As mentioned in the introduction, the double knock-out of ApoER2 and VLDLR leads to a Reeler/Disabled-like phenotype. While both ApoER2 and VLDLR are present in the MZ at E17.5, they typically do not co-localize in the same neurons [10]. In the MAZ, however, VLDLR is absent whereas ApoER2 is mainly localized to neuronal processes and the cell membranes of multipolar cells. These different expression patterns may contribute to the distinct actions of Reelin on migrating neurons during the development of the cerebral cortex. This is also reflected by the fact that single knockouts of either one of the receptors exhibit milder and divergent phenotypes [2], and that ApoER2 signaling is crucial for neuronal migration of late born neurons in the cortex, whereas VLDLR is essential for the termination of migration [12].

**Figure 2:**
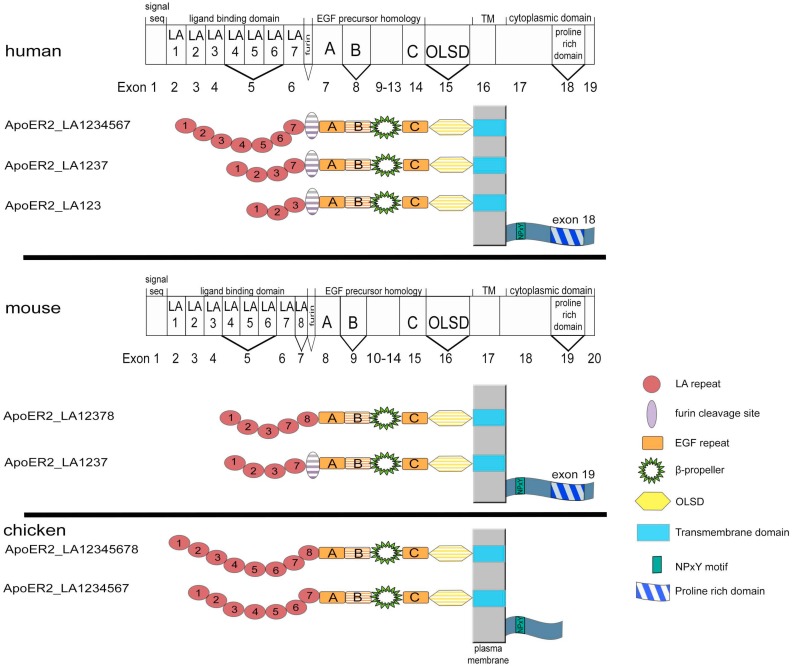
ApoER2 variants present in human, mouse, and chicken. ApoER2 transcript/exon organizations for human and mouse are presented as rectangular bars with exon numbers noted below. Corresponding protein domains are depicted inside the boxes (LDL receptor type A repeats, LA; epidermal growth factor precursor-like repeats, A–C; *O*-linked sugar domain, OLSD; proline-rich region). Structural domains that are not corresponding to single exons are delineated above the rectangular bar (transmembrane domain; TM). Alternatively spliced exons are indicated by V. Domain-structures of the corresponding proteins are shown below the gene structure. Hatched domains can be deleted from receptor variants by alternative splicing. Domains are not drawn to scale.

The situation that there are two highly homologous receptors present for Reelin, which can act together or independently is even more complex since both of the receptors can be expressed in many different variants by alternative splicing of the corresponding genes. Here, we present a comprehensive list of these variants that were detected in different species (Figure 2). Interestingly, the human gene for ApoER2 significantly differs from the chicken and murine genes in that it lacks the exon encoding LA-repeat 8 [13,14]. Splice variants of ApoER2 were studied in human, mouse, and chicken [7,15,16].

In humans, varying transcripts encode the extracellular domain. The full length receptor contains 7 LA repeats. The predominant form lacks exon 5, encoding LA repeats 4–6, and gives rise to a receptor containing LA repeats 1–3 and 7. Another variant contains only LA repeats 1–3 [16]. Some transcripts come with an additional exon coding for a furin cleavage site (13 amino acids) located between the last LA repeat and the epidermal growth factor repeats [13]. Additionally, there are variants missing epidermal growth factor repeat B or the *O*-glycosylation domain [7]. The cytoplasmic domain of ApoER2 in mammals facultatively contains a unique proline-rich region composed of 59 amino acids, which is encoded by exon 19 in non-primate placental mammals or exon 18 in primates [17]. The problem here is that it was never evaluated which combination(s) of these options are indeed present in selected transcripts.

In murine brain, three variants of the ligand-binding domain have been described [15]: The first contains repeats 1–3, 7, 8 (ApoER2-LA12378), the second contains repeats 1–3, 7 with a furin cleavage site between the last LA repeat and repeat A (ApoER2-LA1237F), and the third contains repeats 1–3 and 7 (ApoER2-LA1237). Exon 5 coding for LA repeats 4–6 is spliced out in all transcripts in mice [18]. Similar to human ApoER2, the murine receptor can also be expressed as differentially spliced variants without extracellular *O*-linked sugar domain (-OLSD, Δexon16) and without the intracellular proline rich region [19].

In chicken, two ApoER2 (originally termed LR8B [4]) transcripts are expressed. The difference between the two variants is the absence/presence of LA repeat 8 [13]. This is an interesting observation from the evolutionary point of view. Ligand binding studies using all LA-repeat variants that are present in chicken and mice revealed that all of these variants bind β-VLDL with a similar affinity, while the ability to bind α_2_-macroglobulin depends on the presence of the 8th ligand binding repeat. Evaluation of functional differences in the binding properties of murine LA-repeat variants to Reelin revealed that the presence of the 8th LA repeat interferes with binding of the central Reelin fragment to the receptor [20]. From these observations, we can deduce that chicken ApoER2 may be poorly suited to perform as Reelin receptor (this idea, however, was never experimentally tested). Thus, by the loss of the exon for LA repeat 8 in the human gene and by expressing a differentially spliced variant in mice lacking LA repeat 8, the function of ApoER2 might have shifted from a α_2_-macroglobulin receptor to a specific receptor for Reelin. Since there was a switch from an outside-inside layering of the cortical plate in non-mammalian amniotes to an inside-outside layering in mammals [17], we suggest that this shift in binding specificity might have coincided with this switch.

As described above, the cytoplasmic domain of ApoER2 in mammals contains a unique proline-rich region composed of 59 amino acids (Figure 1). This region is not present in any other member of the LDL receptor family. It contains two potential SH3 binding motifs, PXXP (two prolines spaced by two other amino acids), which suggests a role in signal transduction. Indeed, a two-hybrid screen for potential binding partners led to the identification of JNK-interacting proteins JIP-1 and JIP-2, which act as molecular scaffolds for JNK-signalling and they play essential roles in cell proliferation, differentiation, migration and apoptosis [21,22].

Protein levels of ApoER2 are regulated by E3 ubiquitin ligase IDOL (inducible degrader of the LDLR), which triggers the ubiquitination of the receptor on its intracellular domain leading to degradation in the lysosome [23]. Recently, an interesting study suggested a role of IDOL in regulation of dendritic spine morphogenesis and synaptic plasticity through the modulation of synaptic ApoER2 abundance [24]. As expected, overexpression of IDOL caused decreased levels of ApoER2 which in turn disabled learning and memory formation. Interestingly, constitutive high level of ApoER2 in IDOL-deficient mice also led to defective dendritic spine formation and acute impairment of LTP in hippocampal slices and primary neurons. Thus both, too much or too little of ApoER2 has a deleterious effect and its levels have to be tightly controlled for normal brain function [24]. ApoER2 degradation can be also regulated by the proprotein convertase PCSK9 [25]. Another regulator of ApoER2 trafficking is sorting Nexin 17 (SNX7), which interacts with the cytoplasmic NPxY motif of ApoER2 and facilitates its transport from the early endosomes to the recycling endosomes. Lack of SNX7 led to a defect in Reelin-induced development of the dendritic tree [26,27].

## 3. Structure and Expression of VLDLR

**Figure 3:**
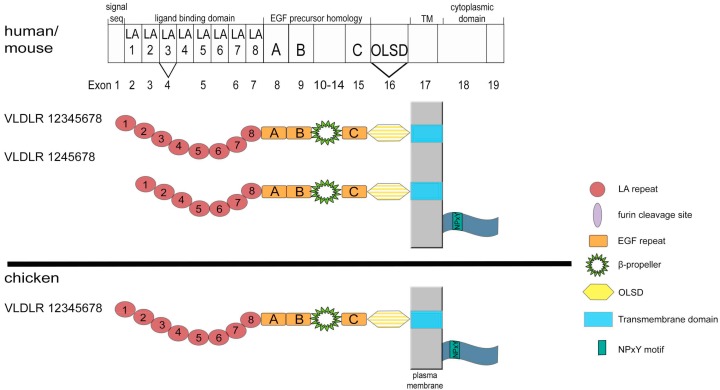
VLDLR variants present in human, mouse, and chicken. VLDLR transcript/exon organizations for human and mouse are presented as rectangular bar with exon numbers noted below. Corresponding protein domains are depicted inside the boxes (LDL receptor type A repeats, LA; epidermal growth factor precursor-like repeats, A–C; *O*-linked sugar domain, OLSD). Structural domains which are not corresponding to single exons are delineated above the rectangular bar (transmembrane domain; TM). Alternatively spliced exons are indicated by V. Domain-structures of the corresponding proteins are shown below the gene structure. Hatched domains can be deleted from receptor variants by alternative splicing. Domains are not drawn to scale.

The first cDNA for the VLDLR was cloned by Yamamoto and colleagues from a rabbit heart cDNA library as a receptor for apoE containing lipoproteins [28]. Corresponding cDNAs from man [29,30], mouse [31], and chicken [32] quickly followed. The VLDLR receptor shows an amazing degree of conservation between different species and its overall structure is almost identical to that of the LDLR (Figure 1); the only difference is the presence of an additional exon coding for an eighth LA repeat [33]. The exon-intron organization of human and mouse genes is completely conserved in these two species (Figure 3) [34]. The human cDNA for VLDLR comprises an open reading frame of 2619 bp (ORF) on 19 exons encoding a protein with 873 amino acids. Like ApoER2, the cDNA for VLDLR can be alternatively spliced, although the produced pattern is much less complex as for ApoER2 (Figure 3). In mammals, VLDLR comes in four potential variants, one as full length form, one lacking the third LA repeat, and possibly both of them missing exon 16, which codes for the *O*-linked sugar domain, all of them are expressed in the mammalian brain [35]. In the retina, only the variant missing the *O*-linked sugar domain is expressed [36]. In chicken oocytes, where VLDLR acts as the key receptor for the uptake of yolk components during oocyte development, the *O*-linked sugar domain is also absent [32]. Interestingly, the OLSD-deficient VLDLR isoform was reported to be more susceptible to proteolytic cleavage indicating a protective role of *O*-linked oligosaccharides against proteases [37,38].

VLDLR expression was confirmed in almost all regions of the central nervous system but the highest amounts were found in the cerebellum and cerebral cortex. It can be found in Cajal-Retzius cells, pyramidal neurons, astrocytes, oligodendrocytes and in neuro- and glioblasts [35]. Outside the brain, high levels of VLDLR are found in skeletal muscles and heart and lower amounts in adipose tissue, kidney, ovary, testis, lung, but not in liver and small intestine [31,39,40,41].

VLDLR expression in the developing cerebral cortex was examined by immunohistochemistry, immunocytochemistry, and in situ hybridization experiments. At embryonic day 14, VLDLR was mainly detected in the MZ. This expression pattern persisted at the later stages. From E16.5, VLDLR was only present in the upper part of the MZ. In reeler mice, VLDLR expression was disturbed. The receptor was not present in the MZ anymore [10]. However, it was dispersed throughout the CP, especially in the internal plexiform zone, which is characteristic for reeler phenotype and is created by abnormal assembly of dendrites [42].

Subcellularly, VLDLR resides in the non-raft fraction of the cell membrane [43]. ApoER2, however, localizes to lipid rafts and this specific sorting is mediated by the extracellular domain of ApoER2. When comparing to VLDLR, ApoER2 bound to Reelin exhibits a much slower endocytosis rate via clathrin coated pit pathway [44].

## 4. Functions of ApoER2

The most prominent function(s) of ApoER2 and VLDLR in mammals are their Reelin receptor activity. This function is prominently demonstrated by ApoER2/VLDLR double knock out mice that exhibit a phenotype indistinguishable from that of Reeler or scrambler mice [2]. Reelin is a large extracellular protein and is the key player in the signaling network involved in establishing laminated structures of the brain (for review see [1,45]. For details about Reelin structure please refer to a recent review [46]. Reelin signaling is tightly regulated by its proteolytic cleavage at two sites, which gives rise to five different fragments (37, 270, 190, 180, and 80 kDa) [47,48,49]. Reelin is secreted as a dimer and Cys 2101 is crucial for the disulfide bond formation; the Reelin mutant lacking Cys2101 is not able to induce downstream intracellular signaling (i.e phosphorylation of Dab1) [50]. Full length Reelin is most abundantly present next to the MZ where it is secreted. Smaller fragments of Reelin can diffuse to deeper layers of the developing brain [47]. 

Binding of Reelin to ApoER2 is mediated by the first LA repeat (LA1) of the receptor and possibly LA2 and LA3 might contribute to additional binding affinity and specificity [51,52,53]. Binding is calcium dependent and can be inhibited by ApoE [54]. As mentioned above, alternatively spliced variants of ApoER2 (ApoER2-LA1237 and ApoER2-LA12378) bind reelin fragments with different affinities and are differentially expressed in a spatio-temporal manner in the developing mouse brain [20].

The isoform carrying four binding repeats and an extra 13 amino acid insertion that contains a furin cleavage site (ApoER2-LA1237F) was shown to produce a soluble ligand binding domain which binds with high affinity to extracellular Reelin and can act in a dominant-negative manner inhibiting Dab1 phosphorylation [18]. Thus, the selective expression of this variant is expected to reduce the Reelin signal in cells expressing this variant. 

ApoER2 can be sequentially processed by α- and γ-secretases [19], similarly to Amyloid precursor protein (APP) or Notch protein [55,56]. The efficiency of processing is modulated by the presence of the *O*-linked sugar domain. A disintegrin and metalloproteinase-10 (ADAM-10) was identified as α-secretase of ApoER2 and APP. TIMP-3, the levels of which are elevated in Alzheimer’s Disease, inhibits the surface expression of ApoER2 and its α-secretase induced cleavage [57]. After the first cleavage, the extracellular part of the receptor is released and the remaining C-terminal fragment (CTF) composed of the transmembrane domain (TM) and intracellular domain (ICD) remains in the plasma membrane. After the second (γ-secretase dependent) cleavage, the soluble ICD is released and translocates to the nucleus and binds to the Reelin promoter, which results in the suppression of Reelin expression at a transcriptional level [58]. ApoER2 processing is enhanced upon Reelin stimulation [59], suggesting that Reelin binding to ApoER2 silences the pathway by decreasing Reelin production. In addition, the ICD of ApoER2, after being translocated to the nucleus, participates in an ApoER2-Reelin-regulated neuronal enhancer complex that is required for the activation of synaptic plasticity genes. ApoER2 proteolysis and the release of ICD seems to be bi-directionally regulated by the activation of NMDAR signaling [60]. The fact that ICD of ApoER2 translocates to the nucleus and acts as gene transcription regulator emphasizes the importance of ApoER2 processing and its regulation. 

As described above, ApoER2 can be expressed as differentially spliced variant without the extracellular OLSD (Δexon16) which contains 36 serine and threonine residues [3]. Knowledge about the function of this isoform is very limited, although in mouse brain it is expressed at similar levels as the full length variant (P. Dlugosz, unpublished observation). Interestingly, there is yet another level of complexity due to this domain. ApoER2 carrying the OLSD comes in two variants; one carrying only N-linked sugar chains (hypo-glycosylated form) and one carrying both, N-linked and *O*-linked sugar chains [61]. Apparently, hyper-glycosylation of full length ApoER2 or loss of the entire OLSD protects against proteolytic cleavage and thus increases surface stability of the receptor. Cells transfected with ApoER2Δexon16 and treated with gamma secretase inhibitor do not produce the CTF, suggesting that the α-secretase cleavage site is within the OLSD. In this situation, multiple bands of varying molecular weights have been produced, which could indicate that ApoER2Δexon16 is processed in a different manner than by the above described sequential cleavage by α- and γ-secretase. ApoER2Δexon16 + 19 (containg exon19; proline rich region; see Figure 1) knock in mice express higher amounts of ApoER2 in the brain. These mice are characterized by increased hippocampal spine density and enhanced hippocampal long-term potentiation (LTP). Surprisingly, this effect did not translate to an increase in associative and spatial learning. Reducing ApoER2 Δexon16 levels to wild type levels by removing one allele of the receptor reversed the enhanced LTP and spine density. These results indicate that ApoER2 Δexon16 plays a role in regulation of synaptic function [61].

**Figure 4:**
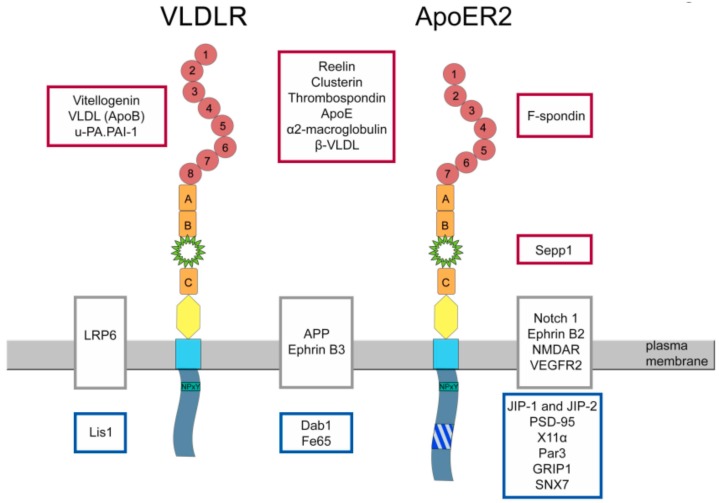
Proteins interacting with VLDLR and/or ApoER2. Proteins interacting only with ApoER2 are indicated in the boxes on the right side of the scheme; proteins interacting only with VLDL receptor are indicated in the boxes on the left side of the scheme; proteins interacting with both receptors are indicated in between the two receptors. Receptor ligands are indicated in the red boxes. Interacting receptors/transmembrane proteins are indicated in the grey boxes. Intracellular adapter proteins are indicated in the blue boxes.

Besides Reelin, there are other ligands which interact with ApoER2 and/or VLDLR (Figure 4). Binding of thrombospondin causes Dab1 phosphorylation, like Reelin does, but without inducing Akt phosphorylation. This signal stabilizes neuronal precursor chains and helps to organize the migration of neuronal precursors from the SVZ to the olfactory bulb [62]. F-spondin binds to ApoER2 and affects the processing of APP [63]. 

ApoER2 and VLDLR were recently identified as receptors for Clusterin (apolipoprotein J) which has been implicated in Alzheimer disease and cancer [64]. Binding of clusterin signals via the Reelin-signaling pathway, through the activation of Dab1, PI3K/Akt, as well as inhibition of cofilin, and stimulates cell proliferation in subventricular zone explants enabling neuronal outgrowth. Possible implications of clusterin as ligand for ApoER2 and VLDLR will be discussed in a later chapter of this review. 

Like VLDLR, ApoER2 can either act as signal transducer or endocytosis competent transport receptor. Selenoprotein P (Sepp1) plays a major role in transporting selenium to organs, like brain, placenta, and testis [65,66]. Selenium is an essential micronutrient and deficiency in the brain leads to irreversible neurological dysfunction and in testis to male hypofertility and structural sperm defects. ApoER2 is the major receptor for Sepp1 (Figure 4), which facilitates Sepp1 endocytosis and selenium supply to the cells [67,68]. In the brain, ApoER2 works as Sepp1 receptor at the blood-brain barrier as well as within the brain on neurons to maintain an extra/intracellular selenium pool which is important to prevent neurodegeneration [69]. Sepp1 binds to the YWTD β-propeller domain of the receptor (Figure 4) and does not require ApoER2 ligand binding repeats that make Sepp1 unique among the other known ligands [70]. Thus, a remnant of the ApoER2-LA1237F that resides in the cell membrane after furin-induced liberation of the soluble ligand binding domain can still act as Sepp1 receptor.

## 5. Proteins Interacting with ApoER2

Interaction of Dab1 with ApoER2 and VLDLR, which is the key event in the Reelin signaling pathway, will not be discussed here in details since recent comprehensive reviews about this pathway are available [71,72]. As mentioned above, the proline rich region in the intracellular domain of ApoER2 interacts with JIP 1 and 2 (Figure 4). A few years after this discovery, PSD-95 (postsynaptic density protein 95) was found to be another binding partner of ApoER2 binding to this region [73]. ApoER2 is present in postsynaptic densities and ApoER2 interacts with NMDA receptor through postsynaptic density protein-95 (PSD-95). Reelin-dependent phosphorylation of Dab1 by Src family kinases further induces these kinases to activate the NMDA receptor by phosphorylating NR2A and NR2B subunits. This process is dependent on the ApoER2 splice variant containing the proline rich region. Reelin-mediated modulation of NMDAR induces Ca^2+^ influx which leads to phosphorylation and activation of cAMP response element-binding (CREB) protein, which serves as transcription factor in activation of learning and memory-associated genes and in the end leads to enhancement of long term-potentiation (LTP). Lack of ApoER2, VLDLR, or Dab1 as well as the inhibition of SFK activity abrogated this neuromodulatory effect of Reelin [74,75]. In addition, Notch1 functionally interacts with ApoER2 and it interconnects with the Reelin pathways in regulating hippocampal plasticity [76].

Mice lacking exon 19 do not express the receptor variant containing the intracellular proline-rich region. They poorly performed in a fear conditioning test (associative learning ability) and in the Morris water maze test (spatial memory) [73]. Although ApoER2 exon 19 is indispensable for the enhancement of LTP in mature brain, it is not required for normal neuronal positioning during embryogenesis. The lamination of cortex and hippocampus in mice constitutively expressing exon 19 or lacking exon 19 did not show any differences when compared with wild type mice. Foliation and cell positioning in cerebellum was unaffected as well. Furthermore, Dab1 and Akt phosphorylation upon Reelin binding did not depend on the presence of exon 19. However, neuronal protection during normal aging by ApoER2 is dependent on the presence of exon 19. This splice variant promotes selective neuronal cell death after injury, which may involve Jun N-terminal kinase (JNK) family [77].

The proline-rich insertion of ApoER2 also binds the adaptor protein X11α (Figure 4) and this interaction increased the migration speed of heterologous cells expressing this ApoER2 variant [78]. Recent studies demonstrated that expression of ApoER2 including exon 19 plays a role in Schwann cell migration by binding PAR3 and activation of Rac1 at the leading edge of these cells [6].

GRIP1 (Figure 4) is another adapter protein interacting with ApoER2 [79]. It bridges a complex, including ApoER2, ephrinB2, and AMPA receptors and regulates the insertion of the AMPA receptor at the synapse. 

ApoER2 and VLDLR are also present on the presynaptic membrane and Reelin induces a transient increase in Ca^2+^ levels in a PI3K-dependent manner. Elevation of Ca^2+^ leads to fusion of vesicles carrying vesicle-associated membrane protein 7 (VAMP7) with an alternative “soluble N-ethyl-maleimide-sensitive factor attachment-protein receptor” (SNARE) protein, which results in a robust neurotransmitter spontaneous release [80].

ApoER2 is thought to be part of a large complex of different receptors in the plasma membrane of neurons (Figure 4). This complex harbors APP [81], Notch [76], Ephrins [82], and NMDAR [83]. Whether such a cluster of different receptors exerts a cooperative function beyond the function of the individual receptors is not known. The recent discovery that ApoER2 directly interacts with VEGF receptor 2 forming a receptor complex that can be activated by either Reelin or VEGF [84] suggests the possibility of such a cooperation. 

## 6. Functions of VLDLR

For VLDLRs general role in the Reelin pathway, we refer again to recent reviews about Reelin and brain development (see above). ApoER2 and VLDLR must be inactivated to recapitulate the Reeler phenotype [2] clearly demonstrating that these receptors have overlapping but distinct functions. The exact interplay of these receptors at the cellular level is still not worked out completely. In the neocortex of VLDLR^−/−^ mice early born neurons (layer V-VI) are located in the inner part of the cortex similar to wild type mice. Late born neurons (layer II-IV) are able to localize to the upper part of the cortex, however, large amount of neurons over migrate and they are positioned in the marginal zone, which indicates that VLDLR is essential for the termination of neuronal migration [12]. Whereas, the cerebellum of VLDLR-deficient mice is small and less foliated with disorganized granule and Purkinje cell layers, the hippocampus appears to undergo normal development [2]. The life span of VLDLR^−/−^ mice is normal and they do not develop recognizable ataxia. Humans, however, lacking VLDLR exhibit a more severe phenotype than mice. Homozygous inactivation of the VLDLR gene in humans results in cerebellar hypoplasia with mild cerebral gyral simplification, which leads to mental retardation, dysarthric speech and cerebellar ataxia [85,86,87], and sometimes quadrupedal gait [87,88]. 

VLDLR was first identified as novel member of the LDLR family that binds apolipoprotein E (Figure 4) with high affinity [28]. Apolipoprotein E binds to the C-terminal part of the LA ligand binding domain and interestingly VLDLR recognizes all apoE isoforms even if there are in a lipid-free state [89]. Reelin binding to VLDLR is mediated by the 5th and 6th Reelin repeat [53].

Due to its apoE-binding properties VLDLR was thought to play a role in systemic lipoprotein homeostasis and in transport of triglyceride-rich apolipoprotein E to cells active in lipid metabolism. However, mice lacking VLDLR did not exhibit elevated plasma levels of triacylglycerol, cholesterol, or lipoproteins, but had a slight decrease in body mass index and adipose tissue mass under normal dietary conditions [90]. Cross-breding of VLDLR^−/−^ mice with LDLR^−/−^ mice led to an increase in serum triglycerides under a high fat diet, suggesting that VLDLR is involved in extrahepatic triglyceride uptake [91]. Another interesting finding was that VLDLR^−/−^ mice on an ob/ob background were protected from obesity under a high fat diet [92]. Forced expression of VLDLR in livers of LDLR^−/−^ mice reversed hypercholesterolemia in these animals, demonstrating that VLDLR has the capacity to clear apoE-containing lipoproteins from the circulation when expressed in the liver [93,94].

As depicted in Figure 4, VLDLR binds many unrelated ligands, such as apoE-containing lipoproteins (see above), urokinase-type plasminogen activator inhibitor complexes [95], thrombospondin [96], vitellogenin [97], apo-E free VLDL [89], and clusterin [64]. 

In chicken and other egg laying species, VLDLR is essential for the uptake of yolk precursors, such as vitellogenin and VLDL (binding of VLDL is mediated by apoB) during the maturation of the oocyte [32,98] and lack of VLDLR causes female sterility [99]. This a very interesting evolutionary aspect of this receptor, since this pivotal role in egg laying species was lost in mammals demonstrated by VLDLR^−/−^ mice that do not show reduction in fertility [90]. 

Defective Wnt signaling has been described in many disorders, e.g., cancer, diabetic retinopathy, diabetic nephropathy, and hypertensive cardiomyopathy [100] and AD [101]. In the retina of VLDLR^−/−^ mice, Wnt signaling is over activated, which leads to the enhanced production of vascular endothelial growth factor and inflammatory factors, and this causes aberrant retinal vascularization and inflammation [102]. VLDLR through binding to LRP6 (Figure 4) which is a co-receptor for Wnts, enhances LRP6 internalization and degradation and therefore negatively regulates Wnt signaling. It was shown that the ectodomain of VLDLR is essential and sufficient for Wnt pathway suppression [103]. Recently, it was shown that the VLDLR variant lacking the OLSD has a higher shedding rate than full length VLDLR and it therefore is more potent in inhibiting Wnt signaling [36].

VLDLR has common intracellular interacting partners with ApoER2 (Figure 4), like Dab1 or FE65 [104]. Lis1, however, belongs to a group of proteins interacting only with VLDLR [105]. 

## 7. Reelin, ApoER2, and VLDLR in Alzheimer’s Disease

Reelin expression levels decrease with age [106] and reduced Reelin expression contributes to cognitive deficits during normal aging [107]. Reelin depletion seems to be an early phenomenon of AD pathology and is detectable long before Aβ pathology becomes evident [108]; although, they are conflicting results concerning this phenomenon (see later). There are few lines of evidence that Reelin is involved in or even has a protective role against development of Alzheimer’s disease (AD). Major hallmarks of AD are extracellular plaques that are composed of amyloid β protein and hyperphosphorylation of microtubule associated tau protein, which leads to intracellular buildup of so-called “neurofibrillary tangles” (for review see [109]). Reelin signaling counteracts the synaptic suppression induced by amyloid β through the activation of Src kinases and the NMDA receptor [110,111]. Reelin also decreases tau phosphorylation through the inhibition of GSK3β and impaired Reelin signaling may lead to tau-hyperphosphorylation [112]. In a transgenic Alzheimer’s disease mouse model, reduction in Reelin-mediated signaling leads to elevated amyloidogenic APP processing and amyloid-β deposition. Additionally, increased tau phosphorylation and enhanced neurofibrillary tangle formation were observed in these mice [113]. Adult inducible reelin knockout mice behave normally without any impairment in learning and memory. However, they were extremely susceptible to amyloid β-provoked synaptic suppression and they had learning and memory deficits despite very low levels of deposited amyloid [114]. Another study revealed that overexpression of Reelin protects against the deleterious effects of amyloid β. Reelin binds to soluble Aβ_42_ and delays amyloid-β fibril formation preventing cognitive loss in a model of AD [115]. Reelin levels present in the cerebrospinal fluid are altered in AD patients [116]. In contrast to reports that Reelin levels are diminished in AD (see above), Reelin mRNA and protein levels were reported to be increased in human AD brains, but Reelin signaling is impaired through amyloid β-mediated disruption of Reelin homodimers [117,118,119]. In aged animals, Reelin was shown to accumulate within amyloid-like deposits and this effect was significantly accerelated in AD mice [120,121]. Its protease, ADAMTS-4 co-localizes in these aggregates with Reelin in WT as well as in transgentic AD mice [122]. Also, the glycosylation pattern of Reelin in the cerebrospinal fluid of Alzheimer disease patients seems to be altered [123]. From studies performed on SH-SY5Y cells treated with Aβ_42_, we know that Aβ increases levels of Reelin expression and alters its glycosylation and processing [124]. These results point to Reelin as potential therapeutic target in AD [125,126,127].

Apolipoprotein E4 (ApoE4) is the major risk factor for sporadic early and late-onset AD (for review see [128]). As mentioned above, ApoE is a ligand for ApoER2 and VLDLR and it diminishes Dab1 phosphorylation [54]. ApoE4 also impairs synaptic plasticity by sequestering ApoER2, NMDAR, and AMPAR in the endosomes reducing their presence at the cell surface. As a result, the ability of Reelin to prevent LTP suppression is severely impaired [129]. It was reported that, in an isoform-specific manner, ApoE4 inhibits LRP1-mediated Aβ clearance from the mouse brain at the blood-brain barrier and shifts it to a slower VLDLR-mediated one [130].

In addition to Reelin itself, gene polymorphism studies point to the possibility that variations of the ApoER2 gene are also associated with development of AD [131]. ApoER2 association to AD was also recently reported by a study showing that ApoER2 trafficking and processing is influenced by mutations in the presenilin 1 gene identified as risk factor for familial AD [132]. Indeed, the processing of ApoER2 is changed in AD patients in a way that less ApoER2-CTF fragments are produced [133]. This observation is in line with results demonstrating elevated TIMP-3 levels in AD [57] (see above). In addition, it was shown that the binding of ApoE4 selectively affects ApoER2 processing leading to reduced production of the CTF [134]. Studies on patients suffering from AD or a murine AD model revealed that the expression of the ApoER2 variant lacking exon 18 in humans or exon 19 in mice coding for the intracellular proline-rich region is higher than in non-AD brains. Their cognitive deficits correlated with the lack of exon 18/19 [135]. The authors of this study proposed a very interesting and innovative therapeutic approach to correct ApoER2 exon 19 splicing by vertebral injections of antisense oligonucleotides that prevent the exclusion of exon 19 and improved learning and memory in a mouse model of AD.

VLDLR involvement in AD was reported in few independent studies [136,137,138]. The most common variant of VLDLR in the developing brain is the one lacking exon 16 [43]. In AD and aged brains, the VLDLR variant including exon 16 (OLSD) was detected in some neurons and satellite glia, as well as in senile plaques [41]. Whether this observation has a functional significance remains to be established. Interestingly, VLDLR-positive microglia were shown to co-localize in AD brains with apoE and amyloid β in senile plaques [139]. VLDLR staining in dentate gyrus was also altered in AD brains. In control brains, staining was uniformly distributed through the entire depth of the molecular layer. In AD dentate gyrus, VLDLR staining was stronger and it appeared mostly in the inner third of this region. Additionally, a stronger expression of VLDLR in AD hippocampal neurons was observed [138].

## 8. Clusterin

As discussed above, Clusterin (CLU) is a recently identified ligand for ApoER2 and VLDLR. Clusterin also called apolipoprotein J is a heterodimeric glycoprotein of 75–80 kDa that is ubiquitously expressed in humans [140]. The gene encodes a 449-aa long protein, which after cleavage of the signal peptide is proteolytically processed into a α- and a β-chain. These two chains become linked in an antiparallel fashion by five disulfide-bonds resulting in an heterodimeric protein that contains three amphipathic helices and two domains characterized by an coiled-coil α-helical structure (for review on structural features of clusterin, see [141]). These structural elements are responsible for clusterin’s “stickiness” i.e., its inherent ability to interact with and bind to many unrelated proteins. The mature protein is extensively glycosylated [142] before secretion into diverse body fluids, such as plasma, semen, milk, urine, and cerebrospinal fluid. In human blood, clusterin levels reach concentrations of 0.03–0.11 g/L [143], and most of it is associated with a dense subfraction of HDL [144]. Clusterin has been implicated in many physiological and pathological processes, such as cancer development, sperm maturation, apoptosis, neurodegeneration and AD, complement regulation, lipid transport, and many more (for reviews see [145,146,147]). Despite its involvement in many seemingly unrelated processes there might be a common denominator in relation to many of these functions. Since its discovery as an extracellular chaperone, compelling evidence has accumulated that a chaperone activity might in fact be the common underlying feature mediating its diverse functions (for review see [148]). Clusterin forms stable and soluble complexes with so-called “client proteins” preventing them from forming toxic aggregates and promoting their clearance by receptor-mediated endocytosis. As demonstrated in a rat model in vivo, complexes of CLU and such client proteins are rapidly taken up by the liver and are degraded in the lysosome [149].

## 9. Clusterin in Alzheimer’s Disease

Genome-wide association studies and whole-exome sequencing have revealed more than 20 loci associated with late-onset AD (reviewed in [150]). Most of these genes have been confirmed by a large meta-analysis including more than 74.000 individuals [151]. Amongst the confirmed loci are variants at the *CLU* locus which was identified already in 2009 to be associated with late-onset AD [152]. In addition, unbiased re-sequencing of all CLU coding exons and regulatory regions in a large cohort of late onset AD patients confirmed rare coding CLU variations to be associated with increased AD risk [153]. Despite this strong association, very little is known about the molecular reason for this effect. CLU is highly expressed in the brain and its expression is increased by inflammation [154] and in AD patients [155]. Moreover, CLU is present in amyloid plaques [154] and associates with Aβ in vitro [156] and it inhibits the aggregation and toxicity of Aβ [157]. Detailed studies on the interaction of CLU with Aβ using single-molecule fluorescence techniques revealed that this interaction takes place at multiple levels during Aβ-aggregation. Primarily CLU seems to prevent monomeric Aβ from oligomerization and second it stabilizes oligomers that dissociate from fibrils inhibiting them from being reorganized into toxic fibrils. Thus, CLU not only reduces the amount of fibrils but also the production of Aβ-oligomers which have been found to be as toxic as the insoluble fibrils [158]. On the basis of the amyloid hypothesis which postulates Aβ deposition as the major force to drive AD-pathology [159] the balance between Aβ production and clearance is an important issue. Indeed, late-onset AD patients have rather insufficient Aβ clearance than increased Aβ production, suggesting that impaired Aβ removal plays a key role in amyloid accumulation [160]. In this respect, the proposal that CLU not only prevents fibril formation but may also participate in the clearance of Aβ from the brain across the blood-brain barrier becomes even more important [161]. The receptor responsible for such a transport seems to be LRP2/megalin [162,163]. However, the role of CLU in AD development is still not completely resolved. Studies on AD-mice lacking CLU report significant fewer fibrillary Aβ-deposits and reduced neuritic dystrophy associated with these deposits [164]. Another study using a similar mouse AD model showed that lack of CLU results in a decrease in Aβ-plaques in brain cortex and hippocampus but a significant increase of these plaques in leptomeningeal vessels and arterioles [165,166]. 

Efforts to identify clusterin receptors led to cell surface receptors belonging to the LDL receptor family. It was demonstrated that LRP2 but not LRP1 is a high affinity receptor for clusterin, and that cells expressing LRP2 internalize and degrade clusterin [167]. However, complexed to cellular debris, clusterin might also be taken up by LRP1 [168]. There might be another potentially powerfull pathway to remove Aβ from the extracellular space in the brain. As outlined above, ApoER2 and VLDLR are expressed by neurons throughout the central nervous system to transmit the Reelin signal. We have recently demonstrated that both receptors bind CLU and that this binding elicits a Reelin-like signal [64]. Besides their function as signal transducers, both receptors have endocytic competence. As established in our laboratory, ApoER2 and VLDLR endocytose cognate ligands by different modes. VLDLR, which resides in non-raft domains of the cell membrane, mediates endocytosis of macromolecules and targets the ligand for degradation via the clathrin-coated pit–clathrin-coated vesicle–endosome pathway to the lysosome with high capacity and efficiency. In contrast, endocytosis via ApoER2, which is sorted to raft domains of the membrane, is slow and less efficient [44]. Thus, VLDLR and to a lesser extent ApoER2 might be strong candidates in the brain to remove extracellular complexes of Aβ and CLU via endocytosis and lysosomal degradation by receptor expressing neurons. In support of this idea and as proof of principle, it has been demonstrated that CLU indeed enhances the uptake and lysosomal degradation of Aβ by F9-teratocarcinoma cells expressing LRP2 [169], and that cells expressing VLDLR are able to take up complexes of clusterin and leptin [170].

## 10. Outlook

As summarized in this review, ApoER2 and VLDLR are taking part in a multitude of different pathways and functional modules. The complexity of the situation is due to many seemingly unrelated observations. First of all, both receptors can act as signal transducers as well as endocytosis competent cargo transporters. Second, both receptors bind many different ligands, some of them binding to both receptors, and some of them are specific for either one receptor (Figure 4). Third, both receptors can act on their own or in combination with each other as it is the case in the Reelin signaling pathway. Last but not least, both receptors interact with other receptors present in the membrane possibly creating signaling platforms with yet unforeseen functions (Figure 4). Such an interaction was recently discovered with VEGF receptor 2 [84]. Reelin stimulation of ApoER2 in combination with VEGFR2 leads to Dab1 phosphorylation and acts as an instructive cue in the endothelium for vascularization in the developing cortex. Since VEGFR2 belongs to a family of highly related receptor tyrosine kinases, it might well be that ApoER2 also interacts with other members of these signaling receptors. Another future aspect of the biology of ApoER2 and VLDLR is the Reelin pathway itself. Advances in the field of nonneuronal Reelin signaling suggest that this pathway might also be involved in the development of other organs as well as recently reviewed [171].
